# Mirror Foot: Surgical Approach for Esthetic and Functional Improvement

**DOI:** 10.1055/s-0042-1742341

**Published:** 2022-02-15

**Authors:** Lucas Almeida Guerra, Jefferson Soares Martins, Igor Matsuy Pacheco Lehnen, Gabriella de Figueiredo Rodrigues, Luiz Fernando Batista Santana

**Affiliations:** 1 Departamento de Ortopedia e Traumatologia, Hospital das Clínicas da Universidade Federal de Goiás/Empresa Brasileira de Serviços Hospitalares (EBSERH), Goiânia, GO, Brasil; 2 Unidade do Sistema Músculoesquelético, Hospital das Clínicas da Universidade Federal de Goiás/Empresa Brasileira de Serviços Hospitalares (EBSERH), Goiânia, GO, Brasil; 3 Grupo de Pé e Tornozelo do Hospital das Clínicas da Universidade Federal de Goiás/Empresa Brasileira de Serviços Hospitalares (EBSERH), Goiânia, GO, Brasil

**Keywords:** congenital foot deformities, bone wires, polydactyly

## Abstract

Mirror foot is a rare congenital anomaly on to the spectrum of complex foot polydactyly. It may occur in isolation or associated with other malformations or genetic syndromes. This is a subject little described in the literature, with few publications on its treatment. We herein report the case of a 4-year-old female patient who presented with a left foot with 8 fingers, without other associated deformities, whose complaints included the impossibility of wearing shoes and social stigma. Radiographically, eight metatarsi with their respective phalanges, five cuneiform bones, and absence of bone deformities in the hindfoot were verified. The surgical approach was chosen in order to promote functional and esthetic improvement, as well as a better adaptation to the use of closed shoes, according to the patient's and family's desire. A dorsal and plantar V incision was performed, with resection of three supranumerary rays, including three central metatarsi with their nine corresponding phalanges, two cuneiform bones, tendons and extra digital nerves, followed by suture of the intermetatarsal ligaments, preserving the fingers with normal appearance, decreasing the width of the foot, and maintaining proper support. The reduction was maintained through transmetatarsal fixation with Kirschner wires. The postoperative period went on with the use of a walking boot and zero load, without complications, with removal o the Kirschner wires and allowing load on the limb after twelve weeks.

## Introduction

Polydactyly is the most common congenital anomaly of the toes, with an incidence of 1.7/1,000 live births, positive family history in about 30% of the cases, and it is up to 10 times more frequent among those of Black ethnicity. 1 Polydactyly is classified as postaxial when the supernumerary ray is on the lateral (fibular) face of the foot, and preaxial when it is located in the medial (tibial) face of the foot. Polydactyly is called complex when there is mirror duplication, be it central, dorsal or of the Haas type, when all fingers are cutaneously fused, with a supernumerary ray that may be pre- or postaxial. 2

Mirror foot is a rare subtype of polydactyly, and few articles 3 4 have been published in the literature on the best approach regarding the surgical treatment. In this complex deformity, the patient usually presents 7 to 8 digits, with duplication of the tarsus bones, and involvement of the bones of the hindfoot is rare. 3 5 It may occur in isolation or associated with other malformations, commonly fibular hemimelia, tibial hypoplasia, mirrored hand, and genetic syndromes, such as Laurin-Sandrow syndrome and Martin syndrome. 3 5

There is no standardization for the treatment of these patients. The planning of the surgical technique is performed individually, with the aim of improving gait function, good adaptation to closed shoes, and gains in the esthetic appearance of the foot, based on excision of the supernumerary ray, tendon transfers and positioning of skin flaps. 3 5 The most normal-looking digits are usually preserved. In the central type of mirror foot, the resection of the middle ray results in very functional and cosmetic feet. 5

In the present paper, we will describe the technique used in the surgical treatment of a patient with a central mirror foot cared for at the Foot and Ankle Outpatient Clinic of the Musculoskeletal System Unit of our institution, presenting the postoperative result. The present work was submitted to the institutional Ethics in Research Committee and authorized.

## Case Report

A four-year-old female patient cared for at the Foot and Ankle Surgery Outpatient Clinic of our hospital with complaints of deformity in the left foot, resulting in pain and in the impossibility of wearing closed shoes. She did not present pain when wearing open sandals or walking barefoot, and had age-appropriate neuropsychomotor development. She did not present other malformations, neither had other cases in the family nor parental consanguinity, and had healthy mother, who was a non-smoker and had no complications during pregnancy.

**Fig. 1:**
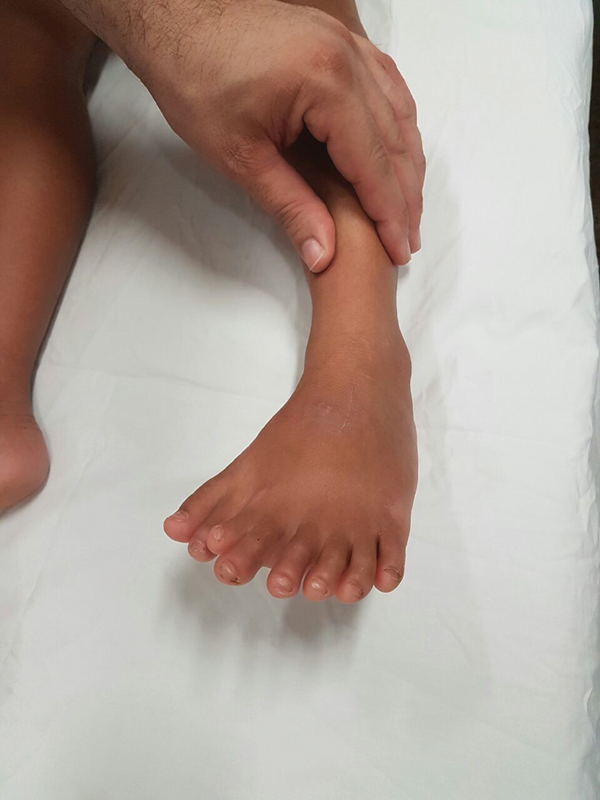
Mirror foot: preoperative clinical aspect.

Upon physical examination, we observed the presence of eight fingers with medialized hallux and central syndactyly ( Figure 1 ), and absence of deformities in the tibia and fibula or shortening of the left lower limb. She presented atypical gait without claudication.

**Fig. 2:**
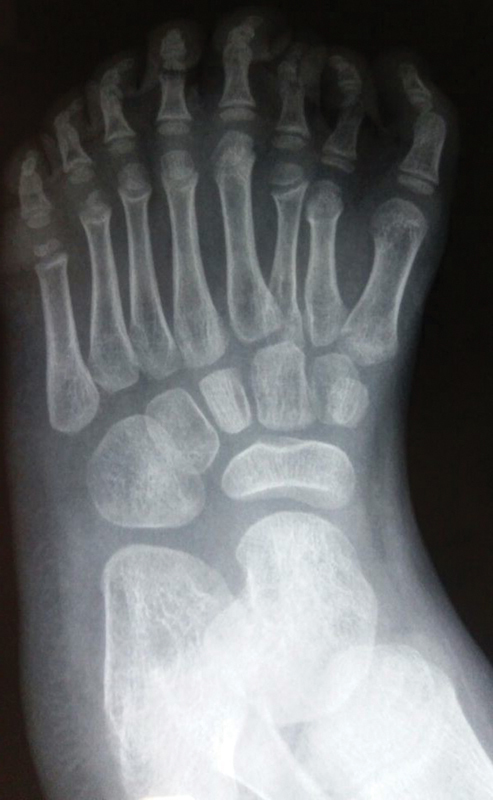
Mirror foot: preoperative radiological aspect.

Radiologically, eight rays with hypoplasia of the third and hypertrophy of the fourth metatarsus were observed, along with the presence of five cuneiform bones and absence of bone fusions in phalanges or deformities in the hindfoot ( Figure 2 ).

### Therapeutic Planning

After the diagnosis of “mirror foot”, the decision for surgical correction was made together with the family and the patient, considering the social repercussions and stigma, the possibility of wearing closed shoes, and the esthetic improvement of the foot.

Surgical planning included the removal of three central supernumerary rays, including three metatarsi with the corresponding nine phalanges, two cuneiform bones and tendons and digital nerves, preserving the fingers that looked closer to normal.

## Description of the Technique

**Fig. 3:**
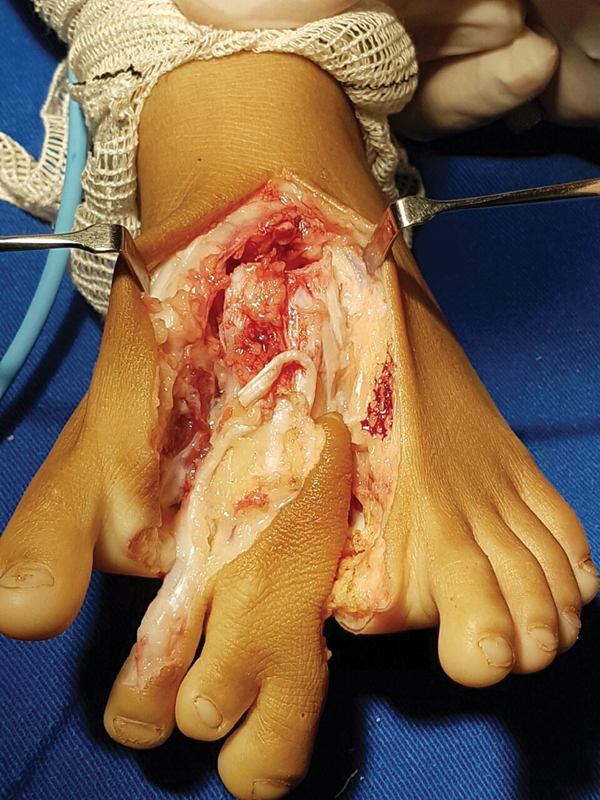
Wedge resection of rays and supernumerary bones.

**Fig. 4:**
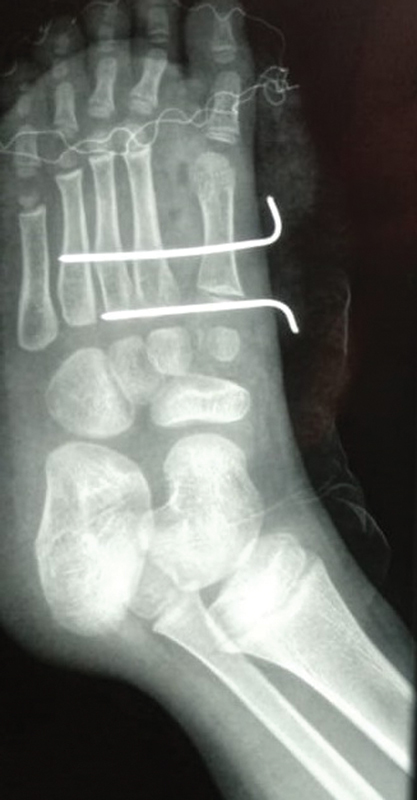
Radiological aspect immediately after the surgery.

**Fig. 5:**
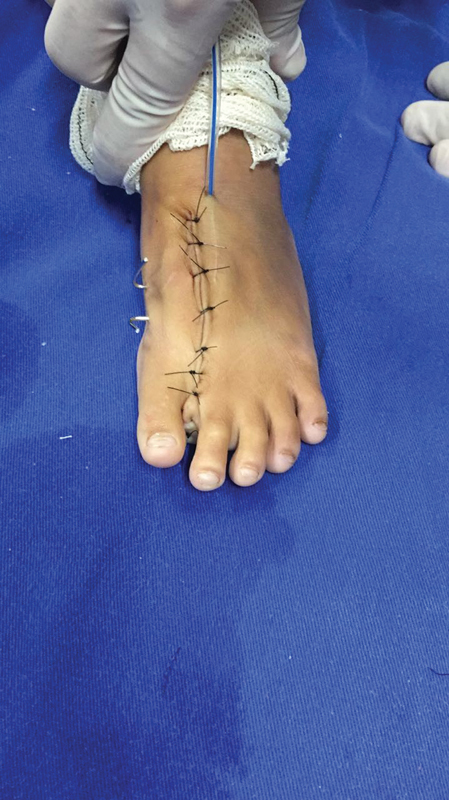
Postoperative clinical aspect.

The procedure was performed in the operating room, with the patient in supine position, under spinal anesthesia, with a lateral cushion and pneumatic tourniquet inflated at 300 mmHg at the level of the thigh. A dorsal and plantar V-shaped skin incision was performed, centered in the middle of the foot ( Figure 3 ), with dissection by planes and resection of the second, third and fourth metatarsi and their second and third cuneiform bones, followed by wedge flap. The removal of the second toe from the medial side meant an inevitable rupture of the Lisfranc ligament. After hemostasis, a reduction in the width of the forefoot was performed, with approximation of the metatarsi and fixation with 2 parallel 1.5-mm Kirschner wires inserted at 90° from the first metatarsus, avoiding transfixing the growth plate. The positioning of the Kirschner wires ( Figure 4 ) was performed with the suture of the intermetatarsal ligaments with absorbable wire. A suction drain was placed, and synthesis by planes was performed using absorbable wire subcutaneously and mononylon 4.0 for skin closure ( Figure 5 ). The drain was removed after 12 hours, with an output of 50 ml of blood.

There is a technical difficulty in the intraoperative period when there is duplication of the cuneiform bones as to how many should be removed and whether the number of metatarsi influences this decision. This removal in bone wedge should be made case by case, and the closure of the forefoot, with consequent reduction in width, should be the main reference parameter for the amount of cuneiform bone to be removed.

There were no intercurrences in the postoperative period, with the patient using a walking boot with zero load. The suture stitches were removed after 18 days, and removal of Kirschner wires occurred after 3 months, when full load was allowed on the limb.

## Discussion

In a survey of the literature published in 2017, Lalé et al. 6 found case reports of 78 patients, totaling 118 mirror feet, and, of these, only 3 cases with normal talus were described. These studies reported a small number of cases, some of them submitted to surgical treatment, but without standardization in the approaches. The strategies adopted in most cases aimed to obtain a functional foot, with satisfactory esthetic presentation.

Central mirror foot is the most unusual form of this rare deformity, comprising only 6% of the cases. 4 We have not found in the Brazilian literature so far any case reports or descriptions of treatments published about this malformation.

Papamerkouriou et al. 4 described the surgical treatment of a case of central mirror foot with dorsal and plantar V-shaped incision in the center of the foot, with removal of the excess skin and three central rays, as well as the extra tendons and digital nerves, similar to what was performed in the case reported in the present technical note.

Shahcheraghi et al. 5 reported the treatment of two cases of central mirror foot in which both had the central rays and excess skin removed, and in one of them the approximation of the other metatarsus was performed by means of cerclage with 1.0-mm steel thread and suture with absorbable thread. In the other case, the approximation of the rays was maintained by inserting a metal pin with the lateral and medial ends bent to provide restraint to the enlargement of the forefoot. In the present work, after the resection of the three central rays and the two excess wedges, we opted for transmetatarsal fixation from the first to the fourth rays with Kirschner wires for stabilization and soft tissue healing.

Vlahovic et al. 8 describe the surgical treatment of a patient with central polydactyly with nine fingers submitted to resection of the four supranumerary rays, with closure of the space generated by fixing the first ray adjacent to the other rays with Kirchner wires, without intercurrences; however, after about 7.5 years, a new intervention was required, due to evolution with adduction of the tarsus bones and varization of the hallux, removing the supranumerary cuneiform bone and correcting the alignment of the first metatarsus and the varus hallux with Kirschner wires.

All of these cases reported in the literature evolved with satisfactory functional and esthetic results, as well as the case herein reported. Allen (1997; apud Osborn et al. 7 ) describes another technique of resection of central rays associated with dorsal and plantar advancement flaps for preservation of the width of the foot, 7 which demonstrated excellent radiographic and functional results in a series of 22 patients and 27 feet with central polydactyly published by Osborn et al. 7

The technique used in the present case encompasses strategies described by these different authors, including: wedge resection of the excess skin and supranumerary bones, closure of the resulting space, and alignment of the remaining rays, aiming at an anatomy close to normal, fixing them with Kirschner wires to preserve the reduction of the intermetatarsal space, achieving good esthetic and functional results. 4 5 8

## Final Comments

Although it is a rare entity with scarce literature, the existing studies have reported good results in the surgical treatment of these patients, promoting better quality of life and satisfaction. The standardization of the most appropriate type of treatment is challenging in view of the rarity and variability of mirror foot in terms of clinical presentation; however, it is evident that one must master the anatomy, and have knowledge of foot biomechanics and concepts of surgical technique for the individualized planning of each case, in the search for restoration of functional and esthetic aspects of the feet affected by this deformity. As a limitation of the present work, the patient was lost to follow-up: she did not return to the outpatient clinic after the allowance of the total load, and could not be reached by telephone, neither in the address registered in the medical records at the beginning of the follow-up.
