# Supplementary material for: Mirror Foot: Surgical Approach for Esthetic and Functional Improvement
Source: Rev Bras Ortop (Sao Paulo). 2022 Feb 15;59(3):e462–6. doi: 10.1055/s-0042-1742341 (PMC11193576; doi:10.1055/s-0042-1742341)
Supplement: Related texts

# Research article: Pé em espelho: Abordagem cirúrgica para melhora estética e funcional

**Keywords:** deformidades congênitas do pé, fios ortopédicos, polidactilia

## Abstract

O pé em espelho é uma anomalia congênita rara, pertencente ao espectro das polidactilias complexas dos pés. Pode ocorrer isoladamente ou associado a outras malformações ou síndromes genéticas. Trata-se de um tema pouco descrito na literatura, com escassas publicações acerca do seu tratamento. Relatamos o caso de uma paciente de 4 anos de idade que apresentava pé esquerdo com 8 dedos, sem outras deformidades associadas, cujas queixas incluíam a impossibilidade de usar calçados fechados e estigma social. Radiograficamente, verificou-se a presença de oito metatarsos com suas respectivas falanges, cinco ossos cuneiformes, e ausência de deformidades ósseas no retropé. Optou-se pela abordagem cirúrgica visando uma melhoria funcional e estética, bem como melhor adaptação ao uso de calçados fechados, conforme desejo da paciente e família. Realizou-se incisão em V dorsal e plantar com ressecção de três raios supranumerários, incluindo três metatarsos centrais com suas nove falanges correspondentes, dois ossos cuneiformes, tendões e nervos digitais extras, seguidas da sutura dos ligamentos intermetatarsais, preservando os dedos com aparência normal, diminuindo a largura do pé, e mantendo seu apoio adequado. A redução foi mantida por fixação transmetatarsal com fios de Kirschner. O pós-operatório seguiu com uso de tala bota e carga zero, sem intercorrências, retirando-se os fios de Kirschner e liberando carga no membro após doze semanas.

## Introdução

A polidactilia é a anomalia congênita mais comum dos dedos dos pés, com incidência de 1,7/1.000 nascidos vivos, história familiar positiva em cerca de 30% dos casos, e até 10 vezes mais frequente na raça negra. 1 Classifica-se a polidactilia como pós-axial quando o raio extranumerário está na face lateral (fibular) do pé, e pré-axial quando localizado na face medial (tibial) do pé. A polidactilia é chamada de complexa quando há duplicação em espelho, central, dorsal, ou do tipo Haas, quando todos os dedos estão fundidos cutaneamente, com um raio extranumerário que pode ser pré- ou pós-axial. 2

O pé em espelho é um subtipo raro de polidactilia e poucos artigos 3 4 foram publicados na literatura sobre a melhor abordagem no tratamento cirúrgico. Nessa deformidade complexa, o paciente normalmente apresenta de 7 a 8 dígitos, com duplicação dos ossos do tarso, sendo raro o comprometimento dos ossos do retropé. 3 5 Pode ocorrer isoladamente ou associado a outras malformações, comumente a hemimelia fibular, a hipoplasia tibial, a mão em espelho, e as síndromes genéticas, tais como a síndrome de Laurin-Sandrow e a de Martin. 3 5

Não existe padronização para o tratamento a ser realizado nesses pacientes. O planejamento da técnica cirúrgica é feito individualmente, com o objetivo de melhora da função da marcha, boa adaptação a calçados fechados, e ganhos na aparência estética do pé, a partir da excisão de raios extranumerários, transferências tendinosas, e posicionamento de retalhos cutâneos. 3 5 Os dígitos de aparência mais normais geralmente são preservados. No tipo central do pé em espelho, a ressecção dos raios médios resulta em pés bastante funcionais e satisafatoriamente estéticos. 5

Neste trabalho, descrevemos a técnica utilizada no tratamento cirúrgico de uma paciente com o pé em espelho central, atendida no Ambulatório de Pé e Tornozelo da Unidade do Sistema Musculoesquelético da nossa instituição, e apresentamos o resultado pós-operatório obtido. Este trabalho foi submetido ao Comitê de Ética em Pesquisa de nossa instituição e autorizado.

## Relato do Caso

Paciente de 4 anos, do sexo feminino, atendida no Ambulatório de Cirurgia do Pé e Tornozelo do nosso hospital, com queixa de deformidade no pé esquerdo que resultava em dor e impossibilidade de usar calçados fechados. Não apresentava dor ao uso de sandálias abertas ou ao deambular descalça, e tinha desenvolvimento neuropsicomotor adequado para a idade. Não apresentava outras malformações, nem outros casos na família ou consanguinidade entre os pais, e tinha mãe saudável, não tabagista, e em cuja gestação não houve intercorrências.

No exame físico notava-se a presença de oito dedos com hálux medializado e sindactilia central ( Figura 1 ), e ausência de deformidade em tíbia e fíbula ou encurtamento do membro inferior esquerdo. Apresentava marcha atípica, sem claudicação.

**Fig. 1:** Aspecto clínico pré-operatório do pé em espelho.

Radiologicamente, observava-se a presença de oito raios com hipoplasia do terceiro e hipertrofia do quarto metatarso, presença de cinco ossos cuneiformes, e ausência de fusões ósseas em falanges ou deformidades no retropé ( Figura 2 ).

**Fig. 2:** Aspecto radiológico pré-operatório do pé em espelho.

## Planejamento Terapêutico

Estabelecido o diagnóstico de “pé em espelho”, a decisão pela correção cirúrgica foi tomada em conjunto com a família e a paciente, levando em consideração as repercussões e estigmas sociais, a possibilidade de usar calçados fechados, e a melhora estética do pé.

O planejamento cirúrgico realizado previa a retirada de três raios extranumerários centrais, incluindo três metatarsos com as nove falanges correspondentes, e dois ossos cuneiformes e os tendões e nervos digitais correspondentes, preservando os dedos com a aparência o mais próximo possível à normal.

## Descrição da Técnica

O procedimento foi realizado em centro cirúrgico, com a paciente posicionada em decúbito dorsal, sob raquianestesia, com coxim lateral e torniquete pneumático no nível da coxa inflado a 300 mmHg. Fez-se uma incisão de pele dorsal e plantar em V centrada no meio do pé ( Figura 3 ), dissecção por planos, e ressecção do segundo, terceiro e quarto metatarsos e suas falanges, e do segundo e terceiro ossos cuneiformes, seguidas por retalho em cunha. A remoção do segundo dedo do pé do lado medial significava uma ruptura inevitável do ligamento de Lisfranc. Após hemostasia, foi feita redução da largura do antepé, com aproximação dos metatarsianos e fixação com 2 fios de Kirschner de 1,5 mm paralelos, inseridos a 90° a partir do primeiro metatarsiano, evitando-se transfixar a fise de crescimento. Checou-se o posicionamento dos fios de Kirschner ( Figura 4 ), e procedeu-se com a sutura dos ligamentos intermetatarsianos com fio absorvível. Instalou-sde dreno suctor e fez-se síntese por planos utilizando fio absorvível no subcutâneo e *mononylon* 4.0 para fechamento da pele ( Figura 5 ). Retirou-se o dreno após 12 horas, com débito de 50 ml de sangue.

**Fig. 3:** Ressecção em cunha de raios e ossos extranumerários.

**Fig. 4:** Aspecto radiológico pós-operatório imediato.

**Fig. 5:** Aspecto clínico pós-operatório.

Existe uma dificuldade técnica no intraoperatório quando há duplicação dos ossos cuneiformes, relacionada a quantos devem ser retirados e se a quantidade de metatarsianos influencia nessa decisão. Essa retirada em cunha óssea deve ser feita caso a caso e o fechamento do antepé, com consequente diminuição da largura, deve ser o principal parâmetro de referência para a quantidade de ossos cuneiformes a serem retirados.

No pós-operatório, não houve intercorrências, e a paciente usou tala bota com carga zero. Os pontos de sutura foram removidos após 18 dias, e a remoção dos fios de Kirschner ocorreu após 3 meses, quando foi liberada carga total no membro.

## Discussão

Em levantamento da literatura publicado em 2017, Lalé et al. 6 encontraram relatos de casos de 78 pacientes, totalizando 118 pés em espelho, sendo que, destes, apenas 3 casos foram descritos com tálus normal. Verificou-se nesses trabalhos um pequeno número de casos descritos, alguns submetidos a tratamento cirúrgico, porém sem padronização nas abordagens. As estratégias adotadas, na maioria dos casos, visavam a obtenção de um pé funcional, com apresentação estética satisfatória.

O pé em espelho central é a forma mais incomum dessa rara deformidade, e compreende apenas 6% dos casos. 4 Não encontramos na literatura nacional até o momento relatos de caso ou descrição de tratamentos publicados sobre esta malformação.

Papamerkouriou et al. 4 descreveram o tratamento cirúrgico de um pé em espelho central com incisão em V dorsal e plantar no centro do pé, com remoção da pele excedente e três raios centrais, bem como dos tendões extras e nervos digitais, semelhante ao que foi realizado no caso descrito nesta nota técnica.

Shahcheraghi et al. 5 relataram o tratamento de dois casos de pé em espelho centrais nos quais ambos tiveram os raios centrais e excesso de pele removidos, sendo que em um deles a aproximação dos demais metatarsos foi feita por meio de cerclagem com fio de aço de 1,0 mm e sutura com fio absorvível. No outro caso, a aproximação dos raios foi mantida por meio da inserção de um pino metálico com as extremidades lateral e medial dobradas para oferecerem contenção ao alargamento do antepé. Neste trabalho, após a ressecção dos três raios centrais e das duas cunhas excedentes, optamos pela fixação transmetatarsiana do primeiro ao quarto raios com fios de Kirschner para a estabilização destes e cicatrização de partes moles.

Vlahovic et al. 8 descrevem o tratamento cirúrgico de uma paciente com polidactlia central com nove dedos submetida a ressecção dos quatro raios supranumerários, com fechamento do espaço gerado fixando o primeiro raio adjacente aos demais raios com fios de Kirchner, sem intercorrências; porém, após cerca de 7,5 anos, foi necessária nova intervenção, devido a evolução com adução dos ossos do tarso e varização do hálux, retirando-se o cuneiforme supranumerário e corrigindo o alinhamento do primeiro metatarso e o hálux varo com fios de Kirschner.

Todos estes casos apresentados na literatura evoluíram com resultados funcionais e estéticos satisfatórios, assim como o caso apresentado neste trabalho. Allen (1997, *apud* Osborn et al. 7 ) descreve outra técnica de ressecção de raios centrais associada a retalhos de avanço dorsais e plantares para manutenção da largura do pé, 7 que demonstrou excelentes resultados radiográficos e funcionais em uma série de casos com 22 pacientes e 27 pés com polidactilia central publicada por Osborn et al. 7

A técnica utilizada neste caso engloba estratégias descritas por estes diferentes autores, entre as quais: a ressecção em cunha da pele excedente e dos ossos supranumerários, o fechamento do espaço resultante, e o alinhamento dos raios remanescentes visando uma anatomia próxima à normal, fixando-os com fios de Kirschner para manutenção da redução do espaço intermetatarsal, alcançando-se um bom resultado estético e funcional. 4 5 8

## Comentários Finais

Apesar de se tratar de uma entidade rara, com escassos artigos na literatura, os estudos existentes têm apontado bons resultados no tratamento cirúrgico desses pacientes, promovendo melhor qualidade de vida e satisfação. A padronização do tipo de tratamento mais adequado é desafiadora, tendo em vista a raridade e a variabilidade na apresentação clínica do pé em espelho; contudo, é evidente a necessidade do domínio da anatomia, e conhecimento da biomecânica do pé e de conceitos de técnica operatória para o planejamento individualizado de cada caso, na busca pelo restabelecimento funcional e estético dos pés acometidos por esta deformidade. Como limitação do trabalho, houve a perda do seguimento da paciente, que não retornou ao ambulatório após liberação da carga total, e não pôde ser contatada por telefone ou pelo endereço cadastrados no prontuário no início do seguimento.
